# Zoster-Like Dermatomal Eruption Following Recombinant Zoster Vaccine: A Case Report

**DOI:** 10.7759/cureus.107834

**Published:** 2026-04-27

**Authors:** Reem M AlZahrani, Ahlam H Alsulami, Zubaida H Khan, Amal A Kokandi, Rawabi M Shaikh

**Affiliations:** 1 Medicine, Faculty of Medicine, King Abdulaziz University, Jeddah, SAU; 2 Dermatology, Faculty of Medicine, King Abdulaziz University, Jeddah, SAU; 3 Internal Medicine, King Fahad Armed Forces Hospital, Jeddah, SAU

**Keywords:** herpes zoster reactivation, herpes zoster virus, shingrix vaccine, zoster vaccine, herpes zoster

## Abstract

The reactivation of the varicella zoster virus (VZV) is the cause of herpes zoster (HZ), usually referred to as shingles. Once an individual has been previously infected with chickenpox, the virus stays dormant in their nervous system and may reactivate later in life, leading to HZ. HZ poses a significant burden on the healthcare system due to its high prevalence. Older adults and individuals with compromised immune function are at higher risk of viral reactivation. Vaccination against HZ is a highly effective strategy in preventing viral recurrence and its associated complications. However, recognizing rare post-vaccination zoster-like cutaneous eruptions is essential. In this report, we present a rare instance of a clinically suspected HZ-like eruption in an 85-year-old man who developed an erythematous maculopapular rash with scattered vesicular lesions on his right neck and shoulder one day after receiving a standard dosage vaccine. The patient was treated with valacyclovir for seven days. The immediate recognition of healthcare professionals is the aim of this report to promote early diagnosis and treatment.

## Introduction

Herpes zoster (HZ), commonly known as shingles, is a prevalent infectious disease resulting from the reactivation of latent varicella zoster virus (VZV), which initially causes varicella (chickenpox), typically during childhood. Following primary infection, VZV remains dormant in sensory nerve ganglia and may reactivate later in life, most commonly in individuals aged 50 years or older. Clinically, HZ is characterized by painful, unilateral vesicular eruptions confined to a dermatomal distribution. The risk of reactivation is increased in association with advancing age, immunodeficiency, malignancy, and chronic systemic illnesses [1,2].

HZ represents a significant public health burden due to its frequency and potential complications, including postherpetic neuralgia, secondary bacterial infection, and, in severe cases, disseminated disease. Early recognition and prompt antiviral therapy are essential to reduce disease severity, limit complications, and improve patient outcomes. Consequently, preventive strategies such as vaccination play a critical role in reducing both the incidence and morbidity associated with HZ.

Shingrix is a non-live recombinant zoster vaccine containing the VZV glycoprotein E antigen and AS01B adjuvant system. It was approved by the U.S. Food and Drug Administration (FDA) in 2017 for the prevention of HZ in adults aged 50 years and older and has demonstrated an efficacy of approximately 97% in preventing HZ across age groups in pivotal clinical trials [3]. The vaccine is generally well tolerated, with most reported adverse effects being mild to moderate and including local injection-site reactions and transient systemic symptoms [3,4]. Importantly, HZ reactivation following recombinant zoster vaccination has not been reported in large clinical trials and remains exceedingly rare in post-marketing surveillance, with only a limited number of case reports documented in the literature [5,6]. Although rare cases of HZ reactivation following vaccination have been reported [7,8], more evidence is needed to establish these as confirmed adverse events following immunization (AEFI).

Here, we report a rare instance of a clinically suspected zoster-like dermatomal eruption occurring shortly after recombinant zoster vaccination in an elderly, mostly immunocompetent patient. The purpose of this case study is to raise physician awareness of unusual post-vaccination appearances and to highlight the significance of prompt diagnosis and treatment.

This case report was previously presented as a poster at the 8th SaudiDerm Annual Conference on January 16-18, 2025.

## Case presentation

**Figure 1:**
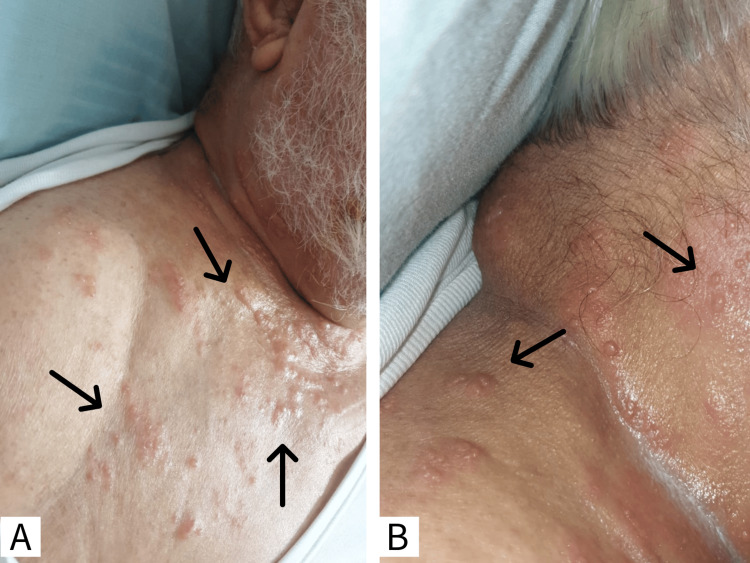
Erythematous maculopapular rash with scattered vesicular lesions over the right side of the A) anterior chest (black arrows) and B) neck (black arrows).

An 85-year-old male patient presented to the hospital with a painful blistering eruption characterized by multiple small vesicles clustered over the right side of the neck, extending to the right shoulder in the C4-5 dermatomal distribution, which appeared one day after receiving the recombinant zoster vaccine, a non-live glycoprotein E subunit vaccine against VZV infection. Prior to the rash onset, the patient experienced fever and malaise. There were no ocular symptoms. The patient had controlled hypertension (valsartan 80 mg and amlodipine), diabetes mellitus (controlled with a balanced diet), retinopathy, neuropathy, and dyslipidemia. He had a history of inguinal hernia repair 20 years prior and an inconclusive history of chickenpox. Based on the clinical diagnosis, the patient was administered valacyclovir (1 g orally three times daily for seven days) and a moisturizing lotion. The next day, he developed pain in the right arm with swelling and redness, which was diagnosed as cellulitis and managed with Augmentin (a combination of amoxicillin and clavulanate). The following day, the patient presented to the emergency department as the swelling increased in the neck and ear pinna. He was febrile on examination. A dermatological assessment showed an erythematous maculopapular rash with scattered vesicular lesions on the right side of the neck, indicating active lesions (Figure 1).

Laboratory tests revealed mild dehydration with normal liver function and lipid profile. No serological or confirmatory diagnostic tests, such as Tzanck smear or viral polymerase chain reaction (PCR), were performed. The diagnosis was made clinically based on the characteristic dermatomal distribution and vesicular morphology in the context of recent vaccination, which represents a limitation of this case report. The patient was treated with paracetamol and ibuprofen alongside ciprofloxacin and linezolid for seven days.

## Discussion

This case highlights a rare occurrence of a zoster-like dermatomal eruption temporally associated with administration of the recombinant zoster vaccine, a non-live adjuvanted subunit vaccine containing glycoprotein E and the AS01B adjuvant system [4]. Glycoprotein E induces VZV-specific immunity, while the AS01B adjuvant enhances both humoral and cell-mediated immune responses through activation of CD4+ T cells and cytokine production [5,6].

The recombinant zoster vaccine in this case contains two immune stimulants: monophosphoryl lipid A (MPL; 3-O-desacyl-4′-monophosphoryl lipid A) and the saponin QS-21, which activate innate immunity and augment antigen-specific antibody and T-cell responses [6]. Although this strong immune activation is essential for vaccine efficacy, it may theoretically contribute to transient immune dysregulation, potentially facilitating viral reactivation in susceptible individuals [6,9].

The most commonly reported systemic adverse effects following recombinant zoster vaccine administration include myalgia, fatigue, headache, fever, shivering, gastrointestinal symptoms, and local injection-site reactions [10]. Importantly, HZ reactivation was not reported in pivotal clinical trials, and cutaneous zoster eruptions remain exceedingly rare in post-marketing surveillance [10]. Nevertheless, a small number of case reports have described dermatomal rashes following this recombinant zoster vaccination.

Published case reports describe similar clinical presentations. A 60-year-old Saudi female patient with type 2 diabetes mellitus, hypothyroidism, and depression developed vesicular eruptions in the L4-L5 dermatomes one week after vaccination [7]. Another report described a 73-year-old female patient with hypothyroidism and hypertension who developed an itchy rash three days after receiving Shingrix [8]. However, in the case reported by Mittal et al. [8], the diagnosis was primarily clinical without laboratory confirmation, highlighting the diagnostic challenges in some reported cases and the importance of confirmatory testing when feasible. These reports are consistent with our findings, suggesting that host-related risk factors may predispose certain individuals to reactivation rather than the vaccine itself [7,8].

Our patient had established risk factors for HZ reactivation, including age above 50 years and diabetes mellitus. Advancing age and metabolic disease are associated with reduced cell-mediated immunity (CMI), which plays a critical role in maintaining VZV latency [9]. Diabetes mellitus, in particular, impairs innate and adaptive immune responses and reduces T-cell-mediated viral control, increasing susceptibility to reactivation [11].

The main strength of this case lies in its contribution to the limited post-marketing safety literature on Shingrix, particularly among patients with metabolic comorbidities. However, limitations include the inability to establish causality, the absence of immunological testing, and reliance on temporal association alone [7,8,10].

Clinically, this case highlights the importance of vigilance rather than contraindication. The recombinant zoster vaccine (Shingrix) continues to demonstrate high efficacy and remains strongly recommended for the prevention of herpes zoster, with its benefits significantly outweighing potential risks [4,10]. Nevertheless, clinicians should provide appropriate counseling to high-risk individuals regarding possible cutaneous adverse events and adopt a proactive approach for early evaluation in the presence of dermatomal pain or rash following vaccination [9,11].

Further studies are needed to clarify the incidence, mechanisms, and risk factors of post-vaccination zoster-like cutaneous eruptions following recombinant zoster vaccination, particularly in patients with impaired immunity or chronic metabolic disease [7-11].

## Conclusions

While rare, the occurrence of zoster-like dermatomal eruptions following recombinant zoster vaccination represents a clinically relevant observation. This case study emphasizes the importance of clinician awareness regarding potential post-vaccination presentations. These findings should not detract from the demonstrated effectiveness of the recombinant zoster vaccine and its significant public health benefits in reducing both the incidence and severity of HZ and its associated complications. Documenting this rare phenomenon holds academic significance as it enhances understanding of post-vaccination immune-mediated responses, stimulates further investigation into vaccine-related immunological mechanisms, and emphasizes the need for vigilant clinical monitoring. Early detection by healthcare providers facilitates timely diagnosis and appropriate intervention, thereby reducing patient-related morbidity. Consequently, this study contributes to vaccine safety literature while promoting enhanced clinical vigilance and improved patient outcomes.
